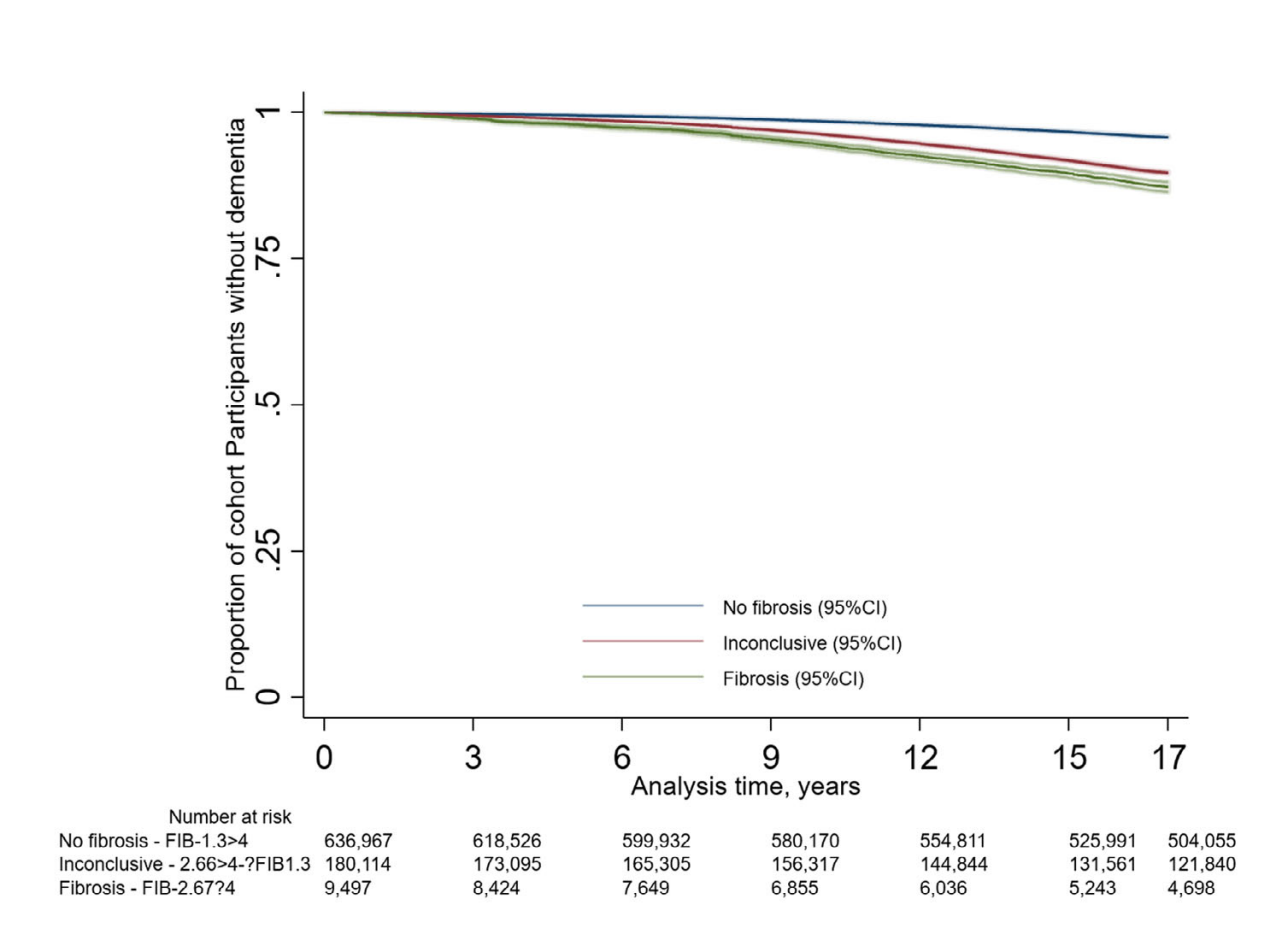# The association between liver fibrosis and dementia risk: a nationwide retrospective cohort study

**DOI:** 10.1002/alz.087316

**Published:** 2025-01-09

**Authors:** Yochai Schonmann, Hanny Yeshua, Shira Zelber‐Sagi, Galit Weinstein

**Affiliations:** ^1^ Tel‐Aviv University, Tel‐Aviv Israel; ^2^ University of Haifa, Haifa Israel

## Abstract

**Background:**

Liver fibrosis is a prevalent, most often clinically silent condition. Accumulating literature suggests that liver fibrosis may be related to brain health measures. However, its predictive role in assessing dementia risk is conflicting. Hence, we assessed the relationship of liver fibrosis with incident dementia in a large, nation‐based cohort.

**Method:**

This retrospective cohort study utilized data from electronic health records of the Clalit Health Services (CHS), the largest health maintenance organization in Israel. We included dementia‐free CHS members aged 40‐69 years who were members for >1 year on January 1^st^, 2006 (baseline). All‐cause dementia was ascertained via ICD‐9 codes, hospital discharge letters and purchase of medications. Liver fibrosis was assessed from recent laboratory measures using the FIB‐4 index, a serum‐based algorithm. Cox regression models were used to assess the relationship between liver fibrosis and dementia incidence while adjusting for various sociodemographic, life‐style and comorbidities. Interaction of liver fibrosis with age, sex, socioeconomic status, ethnicity, obesity, smoking, prevalent diabetes, IHD and history of stroke were further assessed. Lastly, we conducted several sensitivity analyses, among them excluding dementia cases occurring at the first 5‐years of follow‐up.

**Result:**

Of the 826,578 individuals included in the study (mean age 55±8y at baseline), 636,967 (77%) had no fibrosis, 180,114 (21.8%) had inconclusive fibrosis status and 9,497 (1.2%) had high risk for advanced fibrosis according to FIB‐4. Over a mean follow‐up of 15 years, 41,089 dementia diagnoses were recorded. After adjustment for potential confounders, individuals with inconclusive liver fibrosis range and those with advanced fibrosis had a 9% (Hazard Ratio (HR) 1.09, 95%CI 1.07, 1.11) and 18% (HR 1.18 95%CI 1.10, 1.27), respectively, higher dementia risk compared to those with no fibrosis (Figure). The association between liver fibrosis and dementia risk were stronger in those aged 45‐54y compared to other age groups and in persons with no history of diabetes, Ischemic heart disease and stroke. The results remained robust after exclusion of dementia cases occurring in the first years of follow‐up.

**Conclusion:**

Liver fibrosis assessed through a non‐invasive serum‐based algorithm may serve as a novel risk factor for dementia in the general population.